# Orientation of birds in radiofrequency fields in the absence of the Earth’s magnetic field: a possible test for the radical pair mechanism of magnetoreception

**DOI:** 10.1098/rsif.2024.0133

**Published:** 2024-08-07

**Authors:** Jiate Luo, Philip Benjamin, Luca Gerhards, Hannah J. Hogben, P. J. Hore

**Affiliations:** ^1^ Department of Chemistry, University of Oxford, Oxford, UK

**Keywords:** magnetoreception, radical pair, cryptochrome, radiofrequency, compass

## Abstract

The magnetic compass sense of migratory songbirds is thought to derive from magnetically sensitive photochemical reactions in cryptochromes located in photoreceptor cells in the birds’ retinas. More specifically, transient radical pairs formed by light-activation of these proteins have been proposed to account for the birds’ ability to orient themselves using the Earth’s magnetic field and for the observation that radiofrequency magnetic fields, superimposed on the Earth’s magnetic field, can disrupt this ability. Here, by means of spin dynamics simulations, we show that it may be possible for the birds to orient in a monochromatic radiofrequency field in the absence of the Earth’s magnetic field. If such a behavioural test were successful, it would provide powerful additional evidence for a radical pair mechanism of avian magnetoreception.

## 1. Introduction

Although it is clear that migratory songbirds can sense the Earth’s magnetic field as a directional cue [1,2], the underlying biophysical mechanism is largely obscure [3,4]. Arguably the most convincing evidence for a *radical pair mechanism* [5–7] is that birds when tested in Emlen funnels (orientation cages [8]) during the migratory season become disoriented if exposed to weak radiofrequency (1−80 MHz) magnetic fields [9–18]. This diagnostic test for the involvement of radical pairs [19] was inspired by a large number of *in vitro* laboratory studies showing that the yields of organic radical reactions can be modified by time-dependent magnetic fields as well as by static magnetic fields [20–24]. The effect, known as reaction yield detected magnetic resonance (RYDMR), occurs when the oscillating field is in resonance with the coherent interconversion of the singlet and triplet states of the radical pair [20–24]. If the avian magnetic compass sensor is indeed based on the quantum spin dynamics of radical pairs, then RYDMR effects could corrupt the information delivered by the sensor and so lead to disorientation in Emlen funnel tests where the birds’ only source of directional information is the Earth’s magnetic field [25,26].

As in magnetic resonance spectroscopy [27], one would expect a time-dependent magnetic field to have a resonant effect on a spin system when its frequency corresponds to the difference in the energies of two of the eigenstates of the spin system. If all the relevant magnetic interactions are known, it is therefore straightforward to predict the maximum frequency that could elicit a RYDMR response. One simply needs to calculate the difference between the highest and lowest energy eigenvalues of the spin Hamiltonian. This has been done for the flavin–tryptophan radical pair, [FAD^•−‍^ TrpH^•+^], formed in the flavoprotein cryptochrome 4a (Cry4a) that has been proposed as the magnetic sensor in migratory birds [6,7,28–31]. Including the hyperfine interactions of all 27 nuclear spins in the two radicals, together with the electron Zeeman interaction with the Earth’s (approx. 50 μT) magnetic field and the electron–electron dipolar coupling, this maximum frequency is predicted to be 116 MHz [17,18].

In behavioural tests guided by these calculations, Leberecht *et al*. [17,18] found that the ability of Eurasian blackcaps (*Sylvia atricapilla*) to orient in the Earth’s magnetic field could be disrupted by broadband radiofrequency (RF) noise at 75–85 MHz but not at 140–150 MHz or 235–245 MHz suggesting an upper limit or ‘cut-off’ frequency between 80 MHz and 145 MHz consistent with the 116 MHz prediction [17,18]. Taken together with earlier observations of RF disorientation at frequencies below approximately 10 MHz [12,16], these findings provide strong support for a radical pair compass sensor, in which one of the radicals is the semiquinone form of a flavin molecule such as that formed photochemically in Cry4a [31]. They also argue against alternative mechanisms [32] and possible experimental artefacts which would be unlikely to have a pronounced sensitivity fall-off in the range 80–145 MHz [26].

Despite these findings, it is conceivable that the birds’ disorientation could result from an effect of the RF field unconnected to the magnetoreception mechanism (e.g. on the birds’ motivation to orient). Although a devil’s advocate might imagine aspects of the experiments that could lead to *disorientation*, it would be much more difficult to attribute *reorientation* to some strange RF artefact. As far as we are aware, there have been no such reports of RF exposure causing birds to adjust their directional preferences (but see [33] for a possibly related observation in juvenile snapping turtles).

As well as RYDMR experiments involving simultaneously applied static and time-dependent magnetic fields [19–21,24], laboratory studies of photochemically formed radical pairs have also demonstrated that RF fields can alter reaction yields without the need for a static magnetic field [34,35]. If similar effects occurred *in vivo*, the change in the yield of the signalling state could provide the same kind of directional information as a static field and so allow birds to orient in an oscillating field instead of the Earth’s magnetic field. If so, then rotating the axis of the RF field in an Emlen funnel test should cause the birds to prefer a correspondingly rotated compass bearing, an outcome that would provide powerful additional support for a radical pair magnetoreception mechanism.

Here we use spin dynamics simulations to explore this possibility, namely that a radical pair reaction could provide the information necessary for magnetic orientation in an RF field of comparable magnetic flux density to the Earth’s magnetic field.

## 2. Methods

Two methods were used to simulate the effects of static and RF magnetic fields on radical pair reactions. We start by summarizing their common features. The spin Hamiltonian had the form


(2.1) $$\hat{H}\left(t\right)=-\gamma_{e}\left(B_{0}+B_{1}sin\left(2\pi \nu_{RF}t+\gamma \right)\right)\cdot \left({\hat{S}}_{A}+{\hat{S}}_{B}\right)+\sum_{j}{\hat{S}}_{A}.A_{Aj}.{\hat{I}}_{Aj}+\sum_{j}{\hat{S}}_{B}.A_{Bj}.{\hat{I}}_{Bj}+{\hat{S}}_{A}.D.{\hat{S}}_{B},$$

where the vectors $B_{0}$ and $B_{1}$ represent the static and RF fields, respectively, $\nu_{RF}$ and γ are the frequency and phase of the RF field, $A_{Qj}$ is the hyperfine interaction tensor of nucleus *j* in radical Q (∈ A, B), ${\hat{S}}_{Q}$ and ${\hat{I}}_{Qj}$ are, respectively, the electron spin operator for radical Q and the nuclear spin operator for nucleus *j* in radical Q, and **D** is the electron–electron dipolar interaction tensor. Defined in this way, the RF field oscillates along the direction of $B_{1}$ with amplitude varying sinusoidally between +|**B**_1_| and −|**B**_1_|. The yield of the reaction product formed from the singlet state of the radical pair, $\Phi_{S}(\theta )$ , was obtained using the ‘exponential model’ [36] in which singlet and triplet pairs are assumed to react with identical first-order rate constants, *k*, to form distinct products:


(2.2) $$\Phi_{S}\left(\theta \right)=k\int_{0}^{\infty }p_{S}\left(t,\theta \right)e^{-kt}\;dt,$$

where $p_{S}(t,\theta )$ is the singlet probability in the absence of recombination reactions and the angle *θ* defines the direction of the magnetic field axis (**B**_0_ or **B**_1_) with respect to the radicals. For the radical pairs containing the flavin radical, FAD^•−^, the magnetic field axis was restricted to the *zx*-plane of the flavin where the *z*-axis (‍ θ=0 ) is normal to the plane of the tricyclic isoalloxazine group and the *x*-axis ( $\theta =90^{\circ }$ ) is the short in-plane axis (electronic supplementary material, figure S1). In all cases, the radical pair was in a pure singlet state at *t* = 0 and had a lifetime ( $=k^{-1}$ ) of 1 μs, such that an approximately 50 μT magnetic field would have sufficient time to affect the spin dynamics without allowing too much time for spin relaxation (not included) [37]. In the absence of spin relaxation, longer (shorter) lifetimes would increase (decrease) the effects of static and RF magnetic fields to a similar extent. The *relative* change in $\Phi_{S}(\theta )$ , from which one can judge the likely significance of the RF effect, should therefore not depend strongly on the value chosen for the lifetime.

The two methods outlined below have complementary applicability: *γ *-COMPUTE is more efficient for small spin systems, while the approach that uses the stochastic Schrödinger equation works better for large spin systems. We verified that they gave the same results for systems of intermediate size. For radical pairs subject to a static magnetic field, but no RF field, they both agree with simulations performed using the method described by Timmel *et al*. [36]. The Floquet theory approach of Hiscock *et al*. [38] was not used because it treats the RF field as a perturbation, an approximation that is not certainly valid for the approximately 50 μT RF fields of interest here.

### 2.1. *γ *-COMPUTE

The effects of static and RF fields on radical pairs with a small number of hyperfine interactions were calculated using a modified version of the γ -COMPUTE algorithm [39–41] originally devised for simulating solid-state magic-angle spinning NMR spectra of polycrystalline samples. Several changes were required to tailor the method for radical pair reactions [19,24]. First, the periodicity of the spin Hamiltonian comes from the electron Zeeman interactions with the RF field instead of the modulation of anisotropic magnetic interactions by sample spinning. Second, the integral over the powder angle γ becomes an average over the phase, γ , of the RF field. Third, instead of the NMR spectrum, we calculated $\Phi_{S}(\theta )$ using equation (2.1). γ -COMPUTE was used to shed light on qualitative aspects of the RF field effects; for this reason, and for simplicity, the dipolar interaction of the two radicals was not included. Further details of the method are given in [24].

### 2.2. Stochastic Schrödinger equation

To simulate the effects of RF fields on the [FAD^•−^ TrpH^•+^] radical pair, we used the stochastic Schrödinger equation method [42], implemented in the spin dynamics software *MolSpin* [43–45]. The initial nuclear spin space was trace-sampled using 96 *SU*(*Z*) coherent states [42] propagated using the short iterative Lanczos method [46,47] with time steps δt = 0.5 ns or 1 ns for a total time *T* of 5 or 7 μs. Equation (2.2) was used to obtain $\Phi_{S}(\theta )$ with an exponential extrapolation procedure to extend $p_{S}(t,\theta )$ to t=∞ . A total of 14 nuclear spins were included, 7 in each radical (electronic supplementary material, table S1). The dipolar coupling tensor, **D**, was calculated from the separation of FAD and Trp318 in the crystal structure of pigeon Cry4a [47,48]. Despite the name of the method, the calculations presented here were performed for monochromatic RF fields, with no stochastic terms in the spin Hamiltonian.

## 3. Results

### 3.1. Toy radical pair

We start by considering a simple ‘toy’ radical pair containing two nitrogen nuclei (^14^N, spin quantum number, I=1 ‍‍) in one of the radicals and no nuclear spins in the other. The hyperfine tensors were chosen to be axial with co-linear symmetry axes and to have principal components based closely on the nitrogen nuclei at positions N5 and N10 in the flavin adenine dinucleotide radical, FAD^•−^, formed by photoinduced electron transfer in Cry4a [49]:


(3.1) $$A_{1xx}=A_{1yy}=-2.62\;MHz;\;A_{1zz}=49.21\;MHz,$$

(3.2) $$A_{2xx}=A_{2yy}=-0.54\;MHz;\;A_{2zz}=16.94\;MHz.$$

In the absence of external magnetic fields, the spin Hamiltonian in equation (2.1) has nine quadruply degenerate eigenvalues, listed in table 1 (rounded to the nearest 10 kHz).

**Table 1.** Spin energy levels of the toy (2-nitrogen) radical pair.

*n*	1	2	3	4	5	6	7	8	9
$\nu_{n}$ (MHz)	−33.16	−24.75	−16.56	−8.06	0.16	8.55	16.14	24.61	33.08

**Figure 1.**
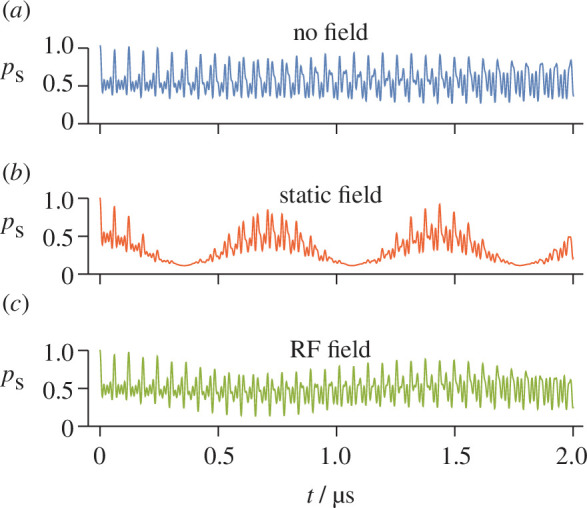
Time dependence of the singlet fraction of a toy radical pair with hyperfine interactions given in equations (3.1) and (3.2) (*a*) with no external magnetic field; (*b*) with a 50 μT static magnetic field; and (*c*) with a 50 μT RF field of frequency 16.6149 MHz. Calculations were performed using the *γ -*COMPUTE method.

Figure 1 shows the probability, $p_{S}(t,\theta )$ , that this radical pair is in a singlet state as a function of time after its formation at t=0 . In this simple example, dipolar coupling, recombination reactions and spin relaxation were all neglected. The three panels correspond to no external field, a $B_{0}$ = 50 μT static magnetic field and a $B_{1}$ = 50 μT RF field with a frequency equal to the difference between the fourth and sixth eigenvalues listed in table 1: $\nu_{RF}=\nu_{6}-\nu_{4}$ . In both figures 1*b* and 1*c*, the magnetic field vector is perpendicular to the symmetry axis of the hyperfine interactions ( $\theta =90^{\circ }$ ‍‍).

In figure 1*a*, we see quantum beats arising from the formation of the radical pair in a coherent superposition state. Singlet–triplet interconversion occurs at frequencies equal to the intervals between the eigenvalues of the hyperfine spin Hamiltonian (equation (2.1)‍ and table 1)‍. These oscillations are still visible in the presence of an Earth-strength static magnetic field (figure 1*b*), but are now superimposed on a slower modulation resulting from the electron Zeeman interaction at the frequency of the Larmor precession in the 50 μT field, i.e. $\gamma_{e}B_{0}/2\pi$ = 1.4 MHz. The change in the time dependence arises because the Zeeman interaction lifts most of the eigenvalue degeneracies and modifies the eigenvectors of the hyperfine Hamiltonian [36,50].

The corresponding calculation for an RF field with peak intensity $B_{1}$ = 50 μT and a frequency in resonance with the $\nu_{6}-\nu_{4}$ transition (16.6149 MHz) is shown in figure 1*c*. The RF field clearly modifies the coherent spin dynamics of the radical pair but not to the same extent as a static field (figure 1*b*). This difference can be understood by noting that the RF field has a major effect on the two energy levels with which it is in resonance, rather than with all nine as is the case for a static field. In addition, the root mean square intensity of the RF field (approximately 35 μT) is smaller than the strength (50 μT) of the static field. Plots similar to figure 1*c* (not shown) were found for RF fields oscillating at frequencies $\nu_{7}-\nu_{3}$ = 32.6983 MHz, $\nu_{8}-\nu_{2}$ = 49.3548 MHz and $\nu_{9}-\nu_{1}$ = 66.2350 MHz (table 1).

**Figure 2.**
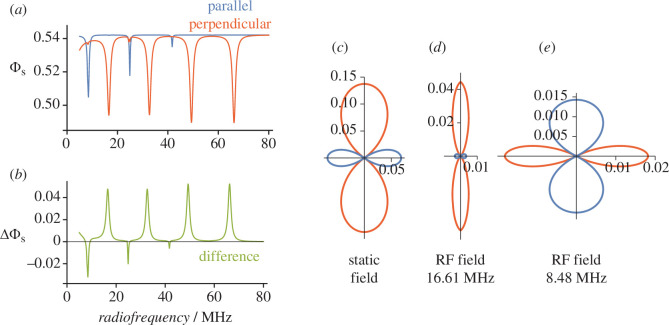
Directional dependence of the singlet yield, $\Phi_{\text{S}}(\theta )$ , of a toy radical pair with hyperfine interactions given by equations (3.1) and (3.2). (*a*) $\Phi_{\text{S}}(\theta )$ as a function of the frequency of an RF field aligned parallel or perpendicular to the hyperfine axis. (*b*) The difference between the two spectra in (*a*). (*c*–*e*) Polar plots of the singlet yield anisotropy, $\Phi_{\text{S}}^{aniso}(\theta )$ , for (*c*) a static field, (*d*) a 16.61 MHz RF field and (*e*) an 8.48 MHz RF field. In each case, the magnetic field strength ( $B_{0}$ or $B_{1}$ ) was 50 μT.

To explore the possibility that a radical pair reaction could extract directional information from an RF field, we used the same toy model to calculate $\Phi_{S}(\theta )$ , the quantum yield of the product formed by spin-selective recombination of singlet radical pairs (equation (2.2)). Figure 2*a* shows the dependence of $\Phi_{S}(\theta )$ on the frequency of a 50 μT RF field aligned parallel ( θ=0 , blue) and perpendicular ( $\theta =90^{\circ }$ , red) to the hyperfine axis. The anisotropy of the magnetic field effect, $\Delta \Phi_{\text{S}}=\Phi_{\text{S}}(0)-\Phi_{\text{S}}(90^{\circ })$ , is shown in figure 2*b*. Four strong resonances appear in the perpendicular spectrum, and three, somewhat weaker and narrower, in the parallel spectrum. As expected, the resonance frequencies correspond to gaps between pairs of the eigenvalues in table 1. Clearly, the influence of the RF field is anisotropic as would be required for a magnetic compass sensor. For comparison, a *static* 50 μT magnetic field under identical conditions gives an anisotropy ( $\Delta \Phi_{\text{S}}$ = 0.206) about four times larger than the maximum RF effect.

The polar plots in figure 2*c–e* show the anisotropy of the singlet yield for a static external magnetic field (with no RF field) and for two RFs (with no static field). In this representation, the spherical average of the reaction yield, $\left\langle \Phi_{\text{S}}\right\rangle$ , i.e. the part of $\Phi_{\text{S}}(\theta )$ that is independent of the magnetic field direction, has been subtracted to reveal the anisotropic component, $\Phi_{\text{S}}^{aniso}(\theta )$ , which contains the directional information. The red and blue colours correspond to positive and negative $\Phi_{\text{S}}^{aniso}(\theta )$ ‍‍, i.e. reaction yields larger and smaller than $\left\langle \Phi_{\text{S}}\right\rangle$ , respectively [37]. Corresponding to the two lowest frequency resonances in figure 2*b*, the polar plots in figure 2*d,e* have opposite signs: an RF field at 16.61 MHz parallel to the hyperfine axis produces a positive $\Phi_{\text{S}}^{aniso}(0)$ while one at 8.48 MHz has a negative effect. The relative sizes of the anisotropic reaction yields can be judged from the values of $\Delta \Phi_{\text{S}}$ : 0.206 for the static field, 0.048 for 16.61 MHz and −0.033 for 8.48 MHz. In this case, other things being equal, the directional signal derived from an RF field on its own is 4–6 times smaller than for a static field.

Based on this toy model, it appears that a radical pair with anisotropic hyperfine interactions subject to a directional RF field could, in principle, deliver the same kind of directional information as it would in a static magnetic field of comparable strength.

### 3.2. Cryptochrome-based radical pairs

**Figure 3.**
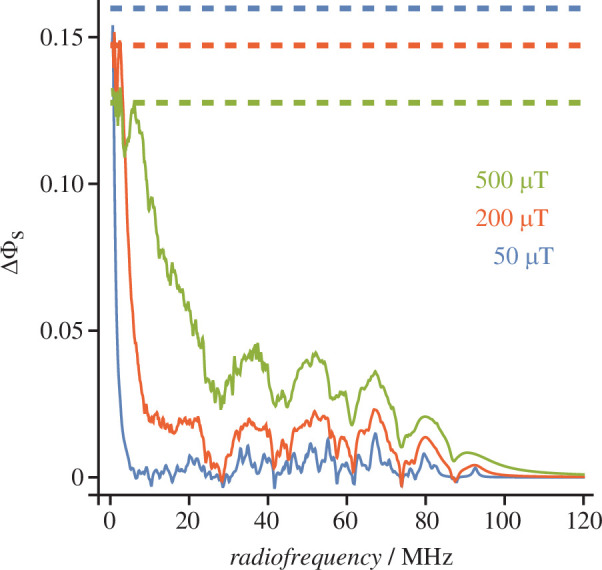
Anisotropy of the singlet yield of a [FAD^•−^ Z^•^] model radical pair calculated for RF peak field strengths *B*_1_ = 50, 200 and 500 μT. $\Delta \Phi_{\text{S}}=\Phi_{\text{S}}(0)-\Phi_{\text{S}}(90^{\circ })$ . The dashed lines are the corresponding values of $\Delta \Phi_{\text{S}}$ for *B*_0_ = 50, 200 and 500 μT static magnetic fields. Calculations were performed using the γ -COMPUTE method.

Having explored the behaviour of an extremely simple spin system, we now turn to more realistic cases based on the flavin–tryptophan radical pairs formed by blue-light excitation of avian Cry4a proteins [31]. To begin, we model a [FAD^•−^ Z^•^] pair [49] in which the Z^•^ radical has no magnetic nuclei, its partner has six of the strongest hyperfine interactions in the FAD^•−^ (the first six entries for FAD^•−^ in table S1 of [49]), and the dipolar coupling, D=0 . Figure 3 shows the anisotropic component, $\Delta \Phi_{\text{S}}$ , of the singlet reaction yield as a function of the frequency of RF fields with $B_{1}$ = 50, 200 and 500 μT. With many more energy levels than the toy spin system, this radical pair has an almost continuous spectrum of resonances running up to approximately 100 MHz, the frequency difference between the highest and lowest energy eigenstates of the hyperfine Hamiltonian. The anisotropy is larger at lower frequencies and for stronger field strengths. For comparison, the dashed lines give the corresponding values of $\Delta \Phi_{\text{S}}$ for static fields of the same intensity. Averaged over the range 20–80 MHz, the anisotropies for 50, 200 and 500 μT fields are, respectively, 2.5%, 8% and 20% of that for a 50 μT static field. That $\Delta \Phi_{\text{S}}$ is larger at frequencies below 10–20 MHz than it is at higher frequencies is probably a result of the ‘Larmor resonance’ at 1.4 MHz which is only expected to occur when (as here) one radical has no (or extremely small) hyperfine interactions and the two electrons have no mutual dipolar or exchange interaction [26].

**Figure 4.**
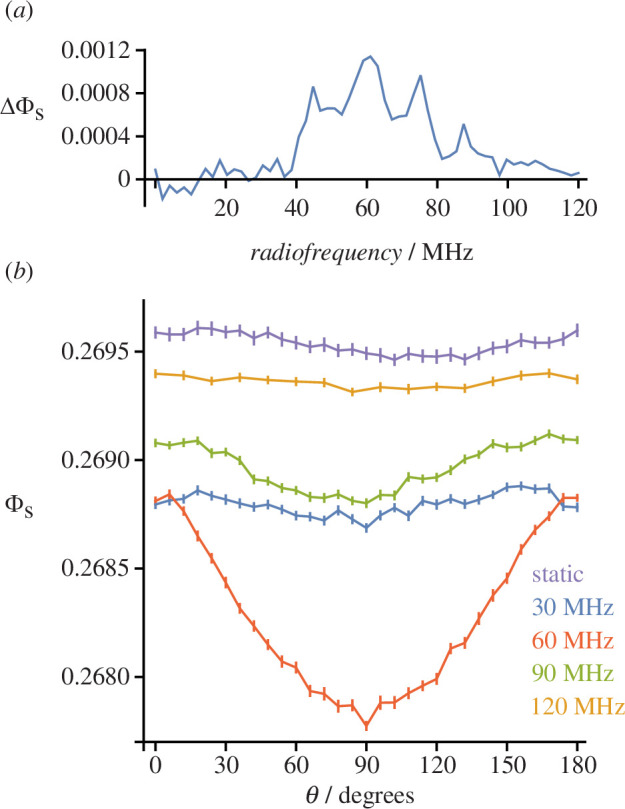
Effects of a 50 μT RF magnetic field on a [FAD^•−‍^ TrpH^•+^] model radical pair. (*a*) $\Delta \Phi_{\text{S}}=\Phi_{\text{S}}(0)-\Phi_{\text{S}}(90^{\circ })$ , as in figure 3. (*b*) $\Phi_{\text{S}}(\theta )$ as a function of the direction of the magnetic field axis for four values of $\nu_{\text{R}\text{F}}$ and for a 50 μT static magnetic field. (*a*) *T* = 5 μs and δt = 1 ns. (*b*) *T* = 7 μs and δt = 1 ns except for 120 MHz where δt = 0.5 ns. The error bars in (*b*) represent the standard error of the mean of the singlet yields for the 96 *SU*(*Z*) states. Calculations were performed using the stochastic Schrödinger equation method.

Finally, we used the stochastic Schrödinger equation approach to simulate a more realistic (16-spin) version of [FAD^•−‍^ TrpH^•+^] comprising a total of 14 nuclei with dipolar coupling included. The results for 50 μT RF fields are shown in figure 4. As expected for such a large spin system [49,51,52], the singlet yield anisotropy $\Delta \Phi_{\text{S}}$ (figure 4*a*) is substantially smaller than for [FAD^•−^ Z^•^]. It peaks at approximately 60 MHz, and lacks the stronger features seen below 10 MHz in figure 3 presumably because there is no Larmor resonance in this case [26]. A speculative explanation for the form of figure 4*a* is given in the electronic supplementary material. The variation of the singlet yield $\Phi_{\text{S}}(\theta )$ with the direction of the 50 μT RF field is shown in figure 4*b* for four values of the RF, $\nu_{\text{R}\text{F}}$ , and for a static magnetic field of the same amplitude. Remarkably, in contrast to figures 2 and 3, the maximum anisotropy in figure 4 is approximately 7 times *larger* for the 60 MHz RF field than for a static field of the same strength. The origin of this difference from the previous calculations is not clear.

## 4. Discussion

Without a doubt, the experiments we have simulated here will be challenging to implement. RF fields with magnetic flux densities of the order of 50 μT will be needed to test whether songbirds can orient in Emlen funnels in the absence of the Earth’s magnetic field. Previous behavioural experiments on RF-induced *disorientation* typically employed much weaker fields: approximately 500 nT at most [9,11,13,14,53,54]. The currents required to produce approximately 50 μT oscillating fields will need greater amplification, which may bring problems with harmonic generation and coil heating. RF fields with the required directional properties can be generated using a single planar coil (e.g. circular or square) [17,18] or, for greater field homogeneity, a Helmholtz pair (two identical planar coils aligned on the same axis, separated by their radius, and carrying equal electric currents in the same direction [14,16]). For the best chance of success, it would probably be wise to match the conditions under which the birds are known to orient in static magnetic fields [9]. That is, the coils should ideally have their axes parallel to the local geomagnetic field which should be nulled by means of direct currents through a separate set of Helmholtz coils or a Merritt four-coil system [55,56]. In principle, the RF coils could surround several Emlen funnels (e.g. [14,16]), although it would probably be more satisfactory to use a separate coil or set of coils for each funnel (e.g. [17,18]). The coil diameter will need to be a compromise between field homogeneity (better with larger coils) and field strength (smaller coils require smaller currents for a given field). The magnetic and electric components of the time-dependent fields should be monitored, for example as recommended in [26] and implemented in [17,18]. Although the simulations discussed here were performed exclusively for single-frequency fields, there may be an advantage in using noise-modulated, broadband RF fields, for example, to cover the 40–80 MHz range suggested by figure 4*a*. In the presence of the Earth’s magnetic field, broadband RF noise seems to have a stronger effect on the birds’ magnetic compass than a single-frequency RF field of the same overall intensity [14]. The same may be true in its absence.

## 5. Conclusion

We have presented spin dynamics simulations of radical pairs subject to directional oscillating magnetic fields. Three cases have been considered—a toy spin system with just two ^14^N nuclei, a heavily truncated version of the flavin–tryptophan radical pair formed in avian cryptochrome 4a (six nuclei in one radical, none in the other) and a considerably more realistic version of [FAD^•−‍^ TrpH^•+^] with electron–electron dipolar coupling and a total of 7 + 7 = 14 nuclear spins. The quantum yields of the reaction products in all three cases were found to depend on the orientation of the radicals with respect to the oscillation axis of the RF field in the absence of a static magnetic field. These calculations establish the principle that a magnetic field oscillating in resonance with the spin dynamics of the radical pair could constitute a magnetic compass sensor comparable to that expected for a static magnetic field. For the most realistic model of the radical pair in Cry4a, our calculations even suggest that a 60 MHz 50 μT RF field could provide a stronger directional signal than the Earth’s (50 μT) magnetic field. If such a behavioural test could be successfully implemented, it would provide powerful additional support for a radical pair mechanism of avian magnetoreception and would argue against the disorienting effects of RF fields being the result of an unknown mechanism unrelated to compass magnetoreception.

## Data Availability

The data that support the findings of this study are shown in figures 1–4. Supplementary material is available online [57].
